# Quantifying site-specific chromatin mechanics and DNA damage response

**DOI:** 10.1038/s41598-018-36343-x

**Published:** 2018-12-27

**Authors:** Daniel B. Whitefield, Stephen T. Spagnol, Travis J. Armiger, Li Lan, Kris Noel Dahl

**Affiliations:** 1 Department of Biomedical Engineering, Carnegie Mellon University, Pittsburgh, PA 15213 USA; 2 Department of Chemical Engineering, Carnegie Mellon University, Pittsburgh, PA 15213 USA; 3 Hillman Cancer Center, University of Pittsburgh Medical Center, Pittsburgh, PA 15232 USA

## Abstract

DNA double-strand breaks pose a direct threat to genomic stability. Studies of DNA damage and chromatin dynamics have yielded opposing results that support either increased or decreased chromatin motion after damage. In this study, we independently measure the dynamics of transcriptionally active or repressed chromatin regions using particle tracking microrheology. We find that the baseline motion of transcriptionally repressed regions of chromatin are significantly less mobile than transcriptionally active chromatin, which is statistically similar to the bulk motion of chromatin within the nucleus. Site specific DNA damage using KillerRed tags induced in loci within repressed chromatin causes an increased motion, while loci within transcriptionally active regions remains unchanged at similar time scales. We also observe a time-dependent response associated with a further increase in chromatin decondensation. Global induction of damage with bleocin displays similar trends of chromatin decondensation and increased mobility only at 53BP1-labeled damage sites but not at non-damaged sites, indicating that chromatin dynamics are tightly regulated locally after damage. These results shed light on the evolution of the local and global DNA damage response associated with chromatin remodeling and dynamics, with direct implications for their role in repair.

## Introduction

The human genome is four gigabases of double stranded DNA wound onto histones to form chromatin with loose spatial organization inside the nucleus^1^. The rheological consequences of this highly entangled polymeric system are impacted by numerous factors including chromatin density and molecular motors^2,3^. Recently, it has been shown that chromatin inside of cells is less binary in its higher-order structure than thought previously: there is a continuum of condensation states with most chromatin existing as 5–24 nm diameter chromatin fibers^4^ rather than more rigidly defined heterochromatin and euchromatin. Thus, to examine the dynamics of chromatin, we employ a system utilizing a human osteosarcoma cell line with a stably incorporated cassette of 96 Tetracycline Response Elements (TREs), named U2OS-TRE, incorporated at a site of heterochromatin near the centromere of the X-chromosome previously described in Lan *et al*.^5^ and Wei *et al*.^6^. TREs are sequences of DNA that allow for control of gene expression through their binding of either Transcription Activator (TA) or Tetracycline Repressor (TetR) proteins^5,6^. TA binding to the TRE leads to transcriptional activation and concomitant chromatin decondensation, and TetR binding to the TRE reinforces transcriptional repression and chromatin condensation^5,6^. While this allows the spatial advantage of examining specific chromatin territories, this method introduces challenges of data analysis from the tracking of a single point within a living cell. We utilize multichannel registration particle tracking algorithms to process images and remove rigid body nuclear motion to track single particle loci, allowing for fiducial image stacks of persistent sub-nuclear motion^2,7^. We find that transcriptionally active regions exhibit chromatin dynamics equivalent to bulk chromatin (as measured by chromatin probes bound inside nucleoli and at telomeres) and transcriptionally repressed regions have reduced mobility consistent with their tight condensation state.

We further explore the impact of DNA damage at different loci by inducing DNA double strand breaks (DSBs) using the KillerRed (KR) fluorescent protein bound to TA and TetR, respectively. KR releases superoxide upon light activation and is known to induce DSBs, among other DNA lesions, locally at the sites of expression^5,6^. DNA damage influences a variety of nuclear functions related to gene expression, replication, and regulation. Many of the molecular factors required for repair of DSBs have been investigated through *in vitro* protein-protein and protein-nucleic acid assays^8^, imaging at the sites of damage in cells modulated through RNAi^9^, and studies of disease models^10^.

Some recent studies have begun to examine the chromatin dynamics of DNA damage and repair. As expected, the nucleosome must be reorganized for DNA to be spliced back together and histones are displaced in response to DSBs^11,12^. Global chromatin mobility within the nucleus after DNA damage has also been studied using particle tracking to investigate the impact of repair proteins^13–15^ or consider different repair pathways^16,17^. Here, we investigate DNA damage within distinct chromatin regions while also comparing chromatin mobility within the rest of the nucleus. We observe that DSBs in transcriptionally repressed regions of chromatin, which typically have reduced mobility relative to bulk or transcriptionally active chromatin, exhibit enhanced dynamics more akin to bulk chromatin following DNA damage induction. Transcriptionally active regions, by contrast, undergo time-dependent changes following DNA damage culminating in chromatin relaxation and reduced force propagation from motor protein activity experienced at these sites, consistent with a physical decoupling of the chromatin network. The resulting effect decreases the probability of large length scale chromatin motion at long timescales, thereby reducing the potential for improper repair and translocations.

## Results

### Measuring chromatin dynamics by bound probes

**Figure 1:**
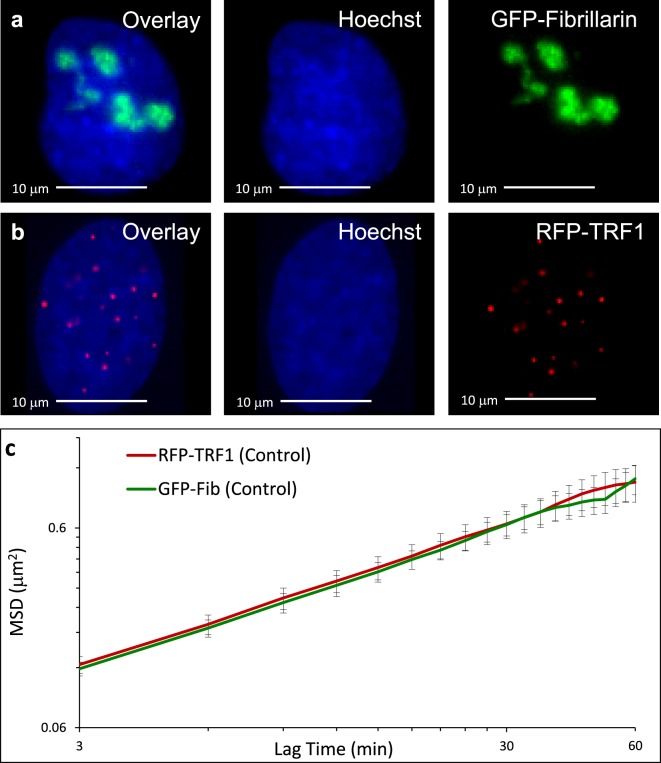
Bulk chromatin motion measurements are independent of bound probe. Nuclei in both cases are stained with Hoechst 33342 (**a**) U2OS cell transfected with GFP-Fibrillarin showing localization of Fibrillarin within nucleoli. (**b**) U2OS cell transfected with RFP-TRF1 showing TRF1 localization at telomeres. (**c**) MSDs of RFP-TRF1 (n = 17), in red, and GFP-Fibrillarin (n = 13), in green, tracked in untreated control U2OS cells. Lines lie atop each other indicating that measurements of MSDs taken from different probes yield equivalent results. Error bars are SEM.

We measure chromatin dynamics inside of nuclei by tracking fluorescently tagged, exogenously expressed, chromatin bound proteins. We have previously demonstrated that ensemble chromatin dynamics on long time scales (minutes) are independent of the chromatin-associated probes. Specifically, in previous work GFP-Fibrillarin and Hoechst 33342 showed indistinguishable mean squared displacements (MSDs)^2^ and GFP-Fibrillarin and GFP-UBF1 were similar to one another; different cell types showed different magnitudes of MSD but a consistency between chromatin-bound probes^7,18^. Theoretically, the mechanics of a viscoelastic polymer solution can be determined from tracking any bound particle in the solution. Here, we track bulk chromatin movements of intranuclear proteins in U2OS human osteosarcoma cells transfected with fibrillarin (GFP-Fibrillarin) or telomeric repeat-binding factor 1 (RFP-TRF1) (Fig. 1a,b). Fibrillarin was chosen because it binds to dense nucleolar regions scattered throughout the nucleus and probes “interstitial” chromatin - chromatin not in close proximity to the end of a chromosome. Conversely, TRF1 was chosen because it specifically binds to telomeres in the nucleus^13^ and probes individual “terminal” chromatin with distinct speckles, allowing for tracking with lower background. Thus, fibrillarin and TRF1 are spatially (Fig. 1a versus 1b) and functionally distinct chromatin moieties. After processing the images to remove rigid body motion of the nucleus and finding centroids of persistent particles, we averaged the MSD of the chromatin bound proteins and plotted these movements versus lag time (Supplemental Fig. 1). For more detailed description of image processing, see the Materials and Methods section or Supplemental Fig. 1. Similar to our other studies, MSDs of GFP-Fibrillarin and RFP-TRF1 are statistically indistinguishable in U2OS cells (Fig. 1c) despite their differential spatial distribution and functional role within the nucleus. We suggest that this similarity in MSD from particle tracking of disparate chromatin-bound probes is consistent with our measurements at these timescales being indicative of ensemble chromatin dynamics of a dense, entangled polymer network. Other work has similarly demonstrated physical mechanisms driving coherent, micrometer-scale chromatin dynamics at short time scales that likely facilitates the physical uniformity of these ensemble chromatin dynamics at our time scales^3^.

### Tracking chromatin motion in transcriptionally active or transcriptionally repressed regions

**Figure 2:**
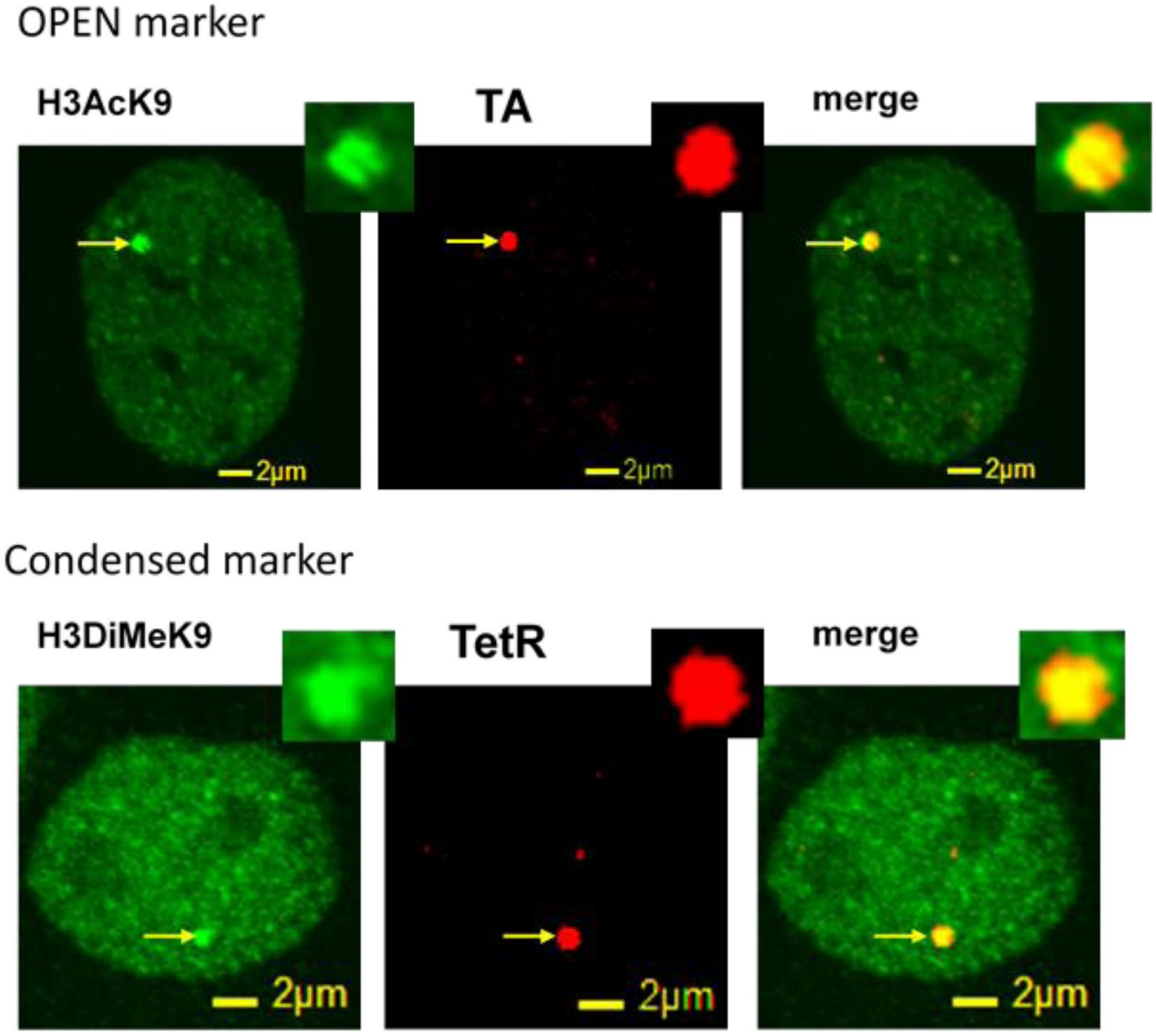
Co-localization of TA and TetR loci with markers of euchromatin and heterochromatin markers, respectively. Co-localization of TA-mCherry with AcH3K9 indicating that the TA motif localizes to regions of euchromatin, and co-localization of TetR-mCherry with DiMeK9H3 indicating that the TetR motif localizes to regions of heterochromatin.

To investigate the distinct chromatin dynamics of transcriptionally active and repressed regions (as opposed to bulk chromatin dynamics) we tracked specific chromatin sites at which we could manipulate transcriptional activity. The effects of heterogeneities of sequences can be diminished with the system. U2OS-TRE has a stably incorporated array of TREs that could be targeted by TetR or TA (TetR + VP16) after transfection to manipulate transcriptional activity and track motion as previously described^5,6^. To show that TA activates transcription we transfected U2OS-TRE cells with Transcription Activated mCherry (TAmCh) and imaged Histone 3 acetylated at Lysine 9 (H3AcK9), a histone modification associated with transcriptionally active regions, by immunostaining (Fig. 2a). Similarly, we transfected U2OS-TRE cells with Tetracycline Repressor mCherry (TetRmCh) and immunostained Histone 3 dimethylated at Lysine 9 (H3DiMeK9), a histone modification that indicates transcriptionally repressed chromatin (Fig. 2b). Additionally, we quantified baseline chromatin dynamics of the TRE array by tracking these regions in live human cell nuclei.

**Figure 3:**
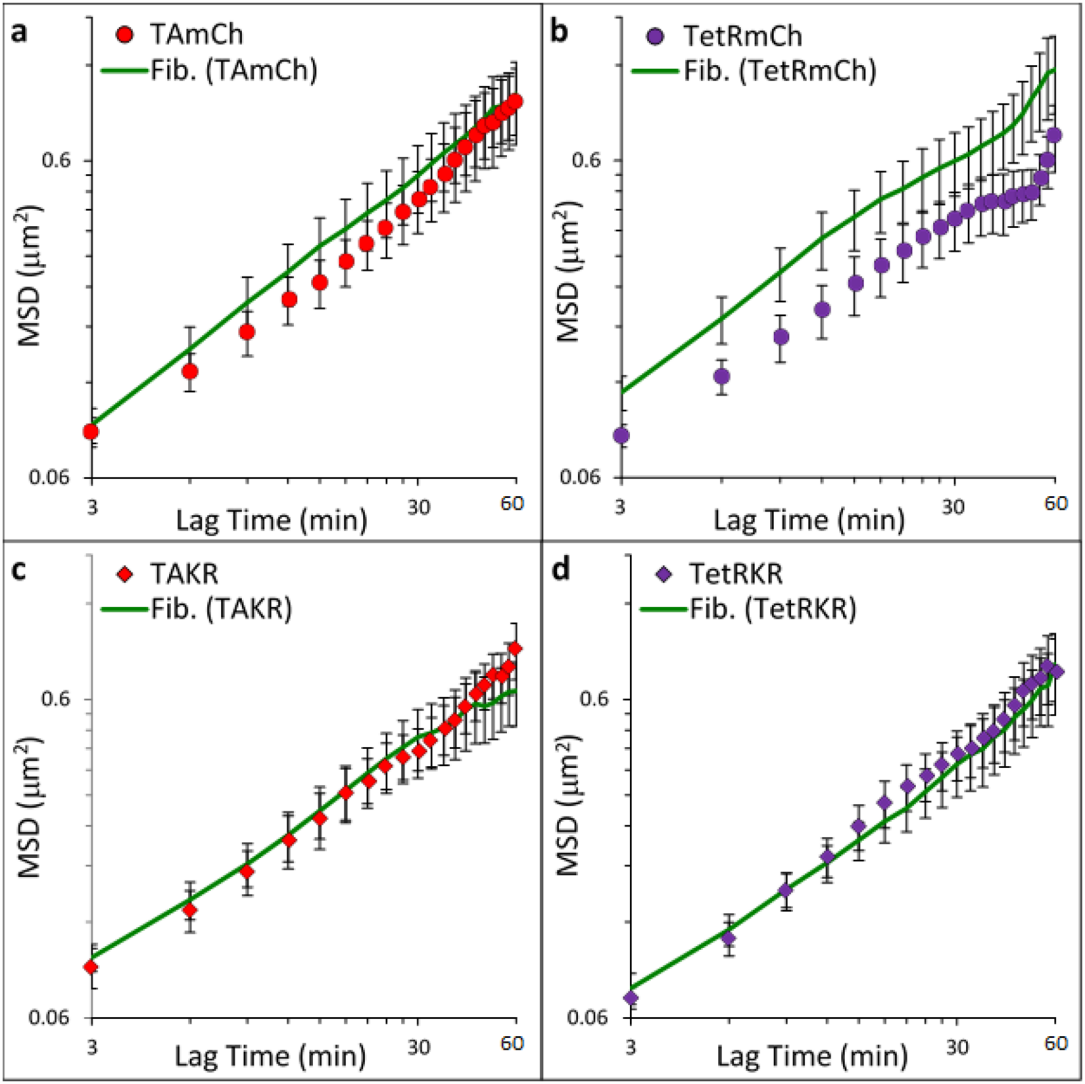
MSDs comparing mobility of the bulk chromatin motion to the four tracers. Bulk chromatin motion measured as cotransfected GFP-Fibrillarin. (**a**) TA-mCherry (TAmCh) denoted by red circles (n = 34), cotransfected GFP-Fibrillarin (Fib. (TAmCh)) denoted by green line (n = 30), (**b**) TetR-mCherry (TetRmCh) denoted by purple circles (n = 27), cotransfected GFP-Fibrillarin (Fib. (TetRmCh)) denoted by green line (n = 31), (**c**) TA-KillerRed (TAKR) denoted by red diamonds (n = 20), cotransfected GFP-Fibrillarin (Fib. (TAKR)) denoted by green line (n = 25), and (d) TetR-KillerRed (TetRKR) denoted by purple diamonds (n = 19), cotransfected GFP-Fibrillarin (Fib. (TetRKR)) denoted by green line (n = 21). Error bars are SEM.

Cells were also co-transfected with GFP-Fibrillarin to visualize the bulk chromatin motion not associated with the TRE array. Our results demonstrate the chromatin dynamics of the bulk network, measured by cotransfected GFP-Fibrillarin and transcriptionally active regions (TAmCh) were indistinguishable (Fig. 3a). By contrast, transcriptionally repressed chromatin regions (TetRmCh) exhibited a significant decrease in mobility from transcriptionally active regions (TAmCh) and the bulk chromatin motion (Fig. 3b) demonstrating that repressed regions are less mobile than transcriptionally active and bulk chromatin motion.

### Tracking DNA damage sites with KillerRed labeled tracer proteins

Since there was a dramatic difference in chromatin dynamics between the transcriptionally repressed regions of chromatin relative to bulk chromatin and transcriptionally active regions of the genome, we explored how DNA damage impacts chromatin dynamics at these sites. To investigate this, we activate KillerRed labeled TA or TetR (in lieu of mCherry) to induce DNA damage at these same TRE regions. Damage induced by TA-KR colocalizes with γH2AX and 53BP1 (Supplemental Fig. 2 and previous studies^5^). Bulk chromatin and transcriptionally active sites had no detectable difference in MSD following KillerRed-induced DNA damage (Fig. 3c). By contrast, chromatin dynamics at transcriptionally repressed (TetRKR) sites were increased (from non-damaged baseline (TetRmCh)) in response to KillerRed-induced DNA damage, now resulting in motion indistinguishable from transcriptionally active (TAKR) and bulk chromatin (Fig. 3d). This observation is consistent with previous work where DSB induction at condensed, transcriptionally repressed regions results in a transition to a more decondensed state^19^, further indicating that site-specific damage also leads to differential local chromatin dynamics.

### Time dependence of chromatin dynamics

**Figure 4:**
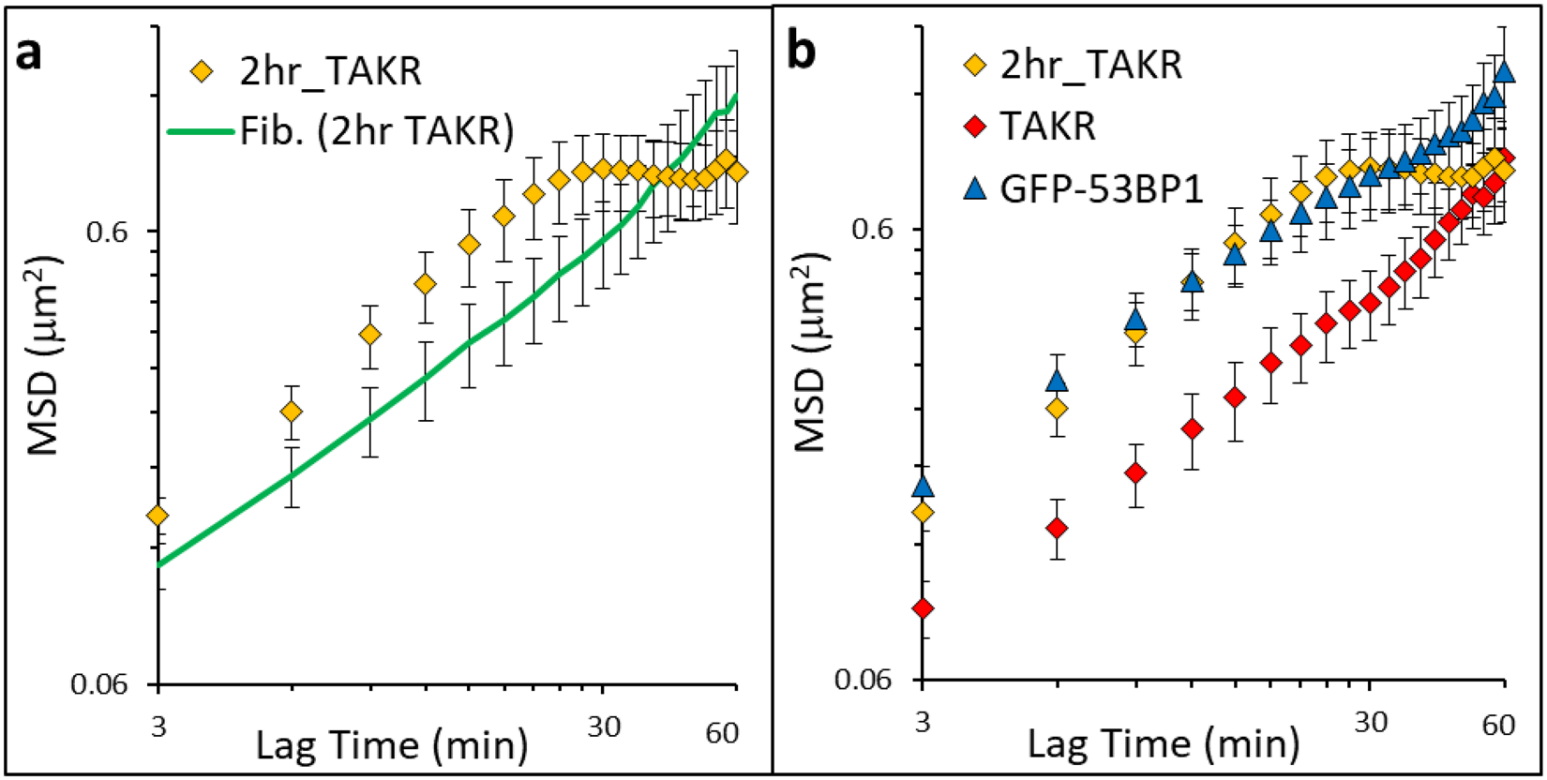
Comparison MSDs showing temporal and global response of chromatin to DNA damage. (**a**) TA-KillerRed after 2 hours (2 hr_TAKR) denoted by orange diamonds (n = 22), cotransfected GFP-Fibrillarin (Fib. (2 hr TAKR)) denoted by green line (n = 26), and (**b**) TA-KillerRed after 2 hours (2 hr_TAKR) again denoted by orange diamonds (n = 22), TA-KillerRed (TAKR) denoted by red diamonds (n = 20), and GFP-53BP1, denoted by blue triangles (n = 12). Mobility is increased after 2 hours. 2 hr_TAKR displays dramatic skew at longer lag times. Error bars are SEM.

Interestingly, DNA damage appeared to impact the chromatin dynamics of transcriptionally repressed, but not at transcriptionally active sites. Given that the majority of transcriptionally active sites are already decondensed for transcription activation, we considered that allowing additional time may change the chromatin dynamics^2^. We cotransfected cells with TAKR and GFP Fibrillarin and induced damage as before, but for this experiment we measured chromatin dynamics after extended time (2 additional hours) post-damage (Fig. 4a). Chromatin at damage foci 2 hours later had increased mobility compared to measurements early after damage, but similar mobility compared to measurements of 53BP1 foci in bleocin treated cells discussed in the next section (Fig. 4b). Unlike other measurements of chromatin, this data showed skew at long lag times and suggesting large variability potentially associated with the presence of DNA lesions other than DSBs due to the high local concentration of reactive oxygen species (ROS).

### Effects of non-specific, nucleus-wide DNA damage

**Figure 5:**
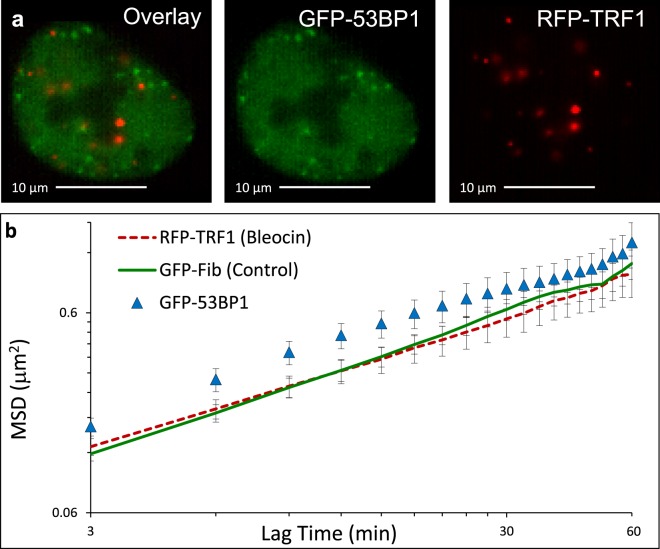
MSDs showing decoupled chromatin dynamics induced by DNA damage. (**a**) U2OS cell treated with bleocin for 2 hours prior to imaging showing the localization patterns exhibited by GFP-53BP1 and RFP-TRF1. (**b**) MSDs from RFP-TRF1 (n = 18) in U2OS cells treated with bleocin, shown in red, and GFP-Fibrillarin (n = 13) in untreated control U2OS cells, shown in green, showing similar mobility to each other despite global damage induction, but increased mobility in damage loci marked by GFP-53BP1 (n = 12), shown as blue triangles. Error bars are SEM. GFP-53BP1 previously used in Fig. 4b. GFP-Fibrillarin (Control) previously used in Fig. 1c.

To measure regions of the chromatin in response to non-specific, nucleus-wide DNA damage, we cotransfected U2OS cells with RFP-TRF1 as well as a protein that binds to double strand breaks: tumor suppressor p53-binding protein 1, GFP-53BP1^20^. We then treated these cells with bleocin for two hours to non-specifically induce nucleus-wide DSBs (Fig. 5a). Following DNA damage induction, the undamaged chromatin sites (labeled with RFP-TRF1 or, in separate experiments, labeled solely with GFP-Fibrillarin following bleocin treatment) show similar MSD to chromatin dynamics of control untreated cells labeled by GFP-Fibrillarin or RFP-TRF1 (Supplemental Fig. 3). Thus, global chromatin MSD appears to be unaffected by the bleocin treatment or fluorescent probe used. However, we observe enhanced chromatin movement in the regions associated with GFP-53BP1, a protein associated with the DNA damage response^21^ (Fig. 5b, triangles). While undamaged chromatin appears capable of maintaining the same behavior as control cells, the movements appear to be different in sites of DNA damage repair shows increased chromatin movements on these time scales, similar to TAKR after extended times (2 hours) but with increased certainty.

## Discussion

The use of particle tracking allows for the characterization of the mechanical microenvironment of the nucleus. The U2OS-TRE system developed by Lan *et al*.^5^ allows for site-specific (in repressed or active chromatin) measurements of chromatin dynamics, as well as allowing for comparison to the bulk chromatin dynamics of the network. To this end, in this study we quantified chromatin dynamics at microenvironments of specific sites of transcriptionally repressed (TetR-mCherry) and transcriptionally active (TA-mCherry) chromatin, then compared these to the bulk chromatin motion (cotransfected GFP-Fibrillarin). This comparison revealed that transcriptionally active chromatin has similar chromatin dynamics to that of the bulk chromatin network. Transcriptionally repressed chromatin, however, displayed reduced mobility compared to that of the bulk average. We previously showed the Hoechst 33342-rich regions, which are higher in heterochromatin content, showed no different mobility than other regions of the chromatin^2^. This is consistent with the idea that the genome is mostly a continuum of chromatin states rather than binary heterochromatin or euchromatin^4^. However, in this case we find a slightly lowered mobility for a singular region that is highly, structurally and functionally compacted.

Replacing mCherry tagged probes with KillerRed tagged probes allowed us to measure changes in mobility after damage had been induced. No significant change could be observed in the mobility of transcriptionally active regions after damage was induced on the order of minutes for a timescale under 2 hours. In transcriptionally repressed regions, however, the mobility increased after damage induction to match that of the bulk mobility of the chromatin network indicating that repressed regions of chromatin become more mobile in response to DNA damage. This observation fits well with previous observations of differential nuclear movements in response to DSBs in heterochromatin versus euchromatin (reviewed nicely in^22^). Recent work in the model *Drosophila* system with distinct heterochromatin and euchromatin regions has shown directionalized movement of heterochromatin after damage^23^. Analysis of our data for preferred directionality did not show any oriented movement despite changes in overall MSD speed (Supplemental Fig. 4), but this difference could also be due to reduced overall movements compared to the *Drosophila* system. It is important to note that since the TRE array is incorporated into transcriptionally repressed regions, even if transcription has been activated by TA and a local region of decondensed chromatin has formed, there may be subtly distinct features displayed by the chromatin dynamics at this induced locus as compared to true endogenous gene expression. Nevertheless, our system provides valuable insight into site-specific differences in chromatin dynamics between transcriptionally active and repressed regions following DNA damage. Previous studies have suggested alternate pathways of DNA damage repair: Homologous Recombination for active chromatin and Non-Homologous End Joining for repressed chromatin^16,17^. While this biophysical study does not consider repair pathways, we observe a relaxation of the repressed chromatin in response to DNA repair processes. This may suggest a minimal fluctuation state of the chromatin or local chromatin territory needed for access of repair factors, despite the pathway used.

We compared chromatin mobility early (microscope experimental setup requires approximately 45 minutes from the completion of damage induction) after ROS induced damage in open chromatin regions (TAKR), after 2 hours (plus microscope setup) of ROS induced damage (2 hr_TAKR), and globally after 2 hours (plus microscope setup) of bleocin induced damage (bleocin with GFP-53BP1). The initial damage did not show increased mobility, but with increasing time chromatin mobility increased compared to the rest of the chromatin in the nucleus. This is an important consideration since the damaged regions are apparently mechanically decoupled from the rest of the chromatin meshwork. This is in contrast to our previous findings, recapitulated here in Fig. 1, that chromatin mechanics can reliably be measured using any other bound probe in any other compartment within the nucleus (telomere, nucleolus, DNA, etc.)^24^.

Previous studies using different imaging modalities and model systems have examined the temporal response to DNA damage and repair. Collectively previous research suggests chromatin decondensation in the first 90 seconds post-damage and recondensation 30 minutes after damage, in some cases to a more compacted state than the native^25,26^. In our experimental setup we do not observe temporal changes, likely because we cannot capture changes before 30 minutes. However, our data provide a useful look into the next steps in these processes. Other work has shown increased chromatin mobility associated with repair of DSBs minutes to hours after damage^13–15,27,28^. Other studies have observed a change in overall coherence of the chromatin after large-scale damage and repair^3,29,30^. Compaction is advantageous early on to signal for certain DDR factors, but, if not reversed, can hinder later stages of repair^26^. Our data reveal that this necessary relaxation occurs over the course of hours following formation of damage foci. Also important to note is that it has recently been shown that chromatin diffusivity is variable when comparing different timescales - i.e. timescales on the order of milliseconds display subdiffusive motion and timescales on the order of seconds display motion closer to Brownian^31^. Our data, taken at timescales on the order of minutes, continues this pattern. Inhibition of proteins in DNA repair pathways (e.g. 53BP1, ATM, SIRT6) have been shown to reduce the increased mobility of chromatin associated with the repair process^13–15^. We speculate that the mechanical decoupling works to prevent large scale movements of the damaged domain so that the free ends of the DSBs remain in close proximity, thereby increasing the likelihood that proper rejoining will occur. Additionally, the local decondensation around the damage foci allows access to repair factors. We also observe that one or many of these mechanically independent mechanical regions may be formed within a single nucleus depending on the number of damaged sites in the nucleus.

## Methods

### Cell Culture, Transfection, and Drug Treatments

The human osteosarcoma cell lines, U2OS and U2OS-TRE^5^, were cultured in DMEM low glucose media supplemented with 10% FBS and 1% penicillin-streptomycin (Life Technologies, Grand Island, NY). Cell cycle was not arrested due to possible alterations in gene expression that could bias results. Instead, imaging was continued for an hour after data collection was complete to ensure cells did not undergo mitosis or apoptosis. U2OS-TRE cells were passaged to 35 mm μ-dishes with ibiTreat (ibidi, Verona, WI) and co-transfected with rDNA of GFP-Fib (kind gift from D. Discher, University of Pennsylvania), and either TA-mCherry, TetR-mCherry, TA-KillerRed, or TetR-KillerRed to visualize chromatin dynamics of various sites^5^. U2OS cells were passaged to 35 mm μ-dishes with ibiTreat (ibidi, Verona, WI) and transfected with rDNA of RFP-TRF1, and GFP-Fibrillarin or GFP-53BP1. Cells were transfected using Lipofectamine 3000 transfection reagent (Life Technologies, Grand Island, NY) according to manufacturer’s protocol. Cells were washed with PBS and media was changed between 5 and 8 hours post transfection, and experiments were run 24–48 hours post transfection to allow for adequate expression levels. For bleocin DNA damage experiments, cells were treated with 5 ng/mL for 2 hours, at which time cells were washed with PBS and media was changed. For U2OS-TRE cells and consistent with previously established methods^5^, photoactivation for all transfection schemes involving TA-mCherry, TA-KillerRed, TetR-mCherry, or TetR-KillerRed was performed using a 15-W SYLVANIA cool white fluorescent bulb for 10 minutes of exposure in a stage UVP (Uvland, CA, USA). This yielded a rate of 15 J/m^2^/s, which, for 10 minutes of exposure, resulted in 9000 J/m^2^ being delivered to the dish and a final power of ~9 nJ delivered to the KR (∼1 μm^2^) upon light exposure. Positive and negative controls for imaging with this system in the absence of white-light illumination were previously published^5^. For late-time experiments with TA-KR transfected cells, the culture dish was returned to the incubator for 2 hours after light exposure before imaging.

### Cell Fixation, Immunostaining, and Colocalization Imaging

Cells in a medium for immunostaining were fixed with methanol-acetone (1:1) for 10 min at −20 °C. The fixed cells were dried, then rinsed once with PBS and incubated in blocking buffer (PBS containing the blocking reagent NEN) at 30 °C for 30 min. Cells were washed three times with PBST (PBS with Tween 20) buffer and incubated with Alexa Fluor 405 goat anti-mouse immunoglobulin G, Alexa Fluor 488 donkey anti-goat immunoglobulin G conjugate or Alexa Fluor 488 donkey anti-rabbit immunoglobulin G conjugate (Invitrogen). Cell samples were then mounted in drops of PermaFluor (Immunon). Antibodies used in this research were anti-KR (Ab961, Evrogen), anti- H3AcK9 (1:200, Abcam Ab4441), anti- H3DiMeK9 (1:100, Abcam 1220). The Olympus FV1000 confocal microscopy system was employed (Cat. F10PRDMYR-1, Olympus) and FV1000 software was used for acquisition of images. Images were acquired with 488 nm and 594 nm, respectively.

### Particle Tracking Imaging and Analysis

Imaging for particle tracking experiments was done using a 63×, 1.4 NA, oil immersion objective of an inverted microscope (DMI6000, Leica, Buffalo Grove, IL) in a controlled live-cell imaging chamber with humidified 5% CO2 and held at 37 °C. Cell nuclei were labeled with 0.5 μg/mL Hoechst 33342 (Life Technologies, Grand Island, NY). Images were taken at multiple (8–12) positions per plate at 3-minute intervals with multiple transfected cells per field of view and multiple particles per cell. Only the bright field, green and/or red channels were acquired with 430–510 nm and 515–560 nm excitation ranges, respectively, for this time to minimize phototoxicity. Cells did not divide and maintained viability well beyond the duration of the experiment as confirmed by continued imaging for over an hour after the completion of data collection. Two-dimensional tracking of GFP-Fib, and either TA-mCherry, TetR-mCherry, TA-KillerRed, or TetR-KillerRed as well as RFP-TRF1 and GFP-Fibrillarin or GFP-53BP1 chromatin regions was performed using custom Laptrack71 programs designed in MATLAB (Natick, MA) as previously published^7,32^. Briefly, images were cropped and aligned to remove artifacts including imaging drift, nuclear translation, and nuclear rotation. Therefore, only intranuclear motion of particles was tracked. Particles were then detected through statistical algorithms after calibration of background noise parameters. Particle tracks were then determined by correspondence with succeeding frames. Only persistent tracks of particles present for the full duration of the experiment were used for further analysis. The ensemble-averaged MSD was calculated from the particle tracks as shown in equation (1) where τ is the lag time.

1 $${\rm{MSD}}\,({\rm{\tau }})=\langle {({{\rm{x}}}_{{\rm{t}}+{\rm{\tau }}}-{{\rm{x}}}_{{\rm{t}}}{)}^{2}+({{\rm{y}}}_{{\rm{t}}+{\rm{\tau }}}-{{\rm{y}}}_{{\rm{t}}})}^{2}\rangle $$

Outliers, defined as tracks which were greater than 3 standard deviations away from the ensemble average at the final lag time, were removed from the dataset. The ‘n’ values reported in figure legends represent the number of cells analyzed. For GFP-Fibrillarin, RFP-TRF1, and GFP-53BP1, each cell may have one or multiple tracks, so the total number of particles tracked is greater than n. For TA-mCherry, TetR-mCherry, TA-KillerRed, and, TetR-KillerRed, since TA and TetR bind to a specific locus within the chromatin, there is only one track per cell, so the total number of particles tracked exactly equals n. MSD magnitudes were compared at each time point using Student’s t-test. Error bars on MSD plots represent Standard Error of the Mean.

## Electronic supplementary material

Supplemental Information

## Data Availability

Data and particle tracking code is available from corresponding author upon reasonable request.
