# Supplementary material for: Quantifying site-specific chromatin mechanics and DNA damage response
Source: Sci Rep. 2018 Dec 27;8:18084. doi: 10.1038/s41598-018-36343-x (PMC6308236; doi:10.1038/s41598-018-36343-x)
Supplement: Supplementary file 1 — Supplemental Information [file 41598_2018_36343_MOESM1_ESM.pdf]

Supplementary Information

Quantifying site-specific chromatin mechanics and DNA damage response

Authors

Daniel B. Whitefield[a,†], Stephen T. Spagnol[b,†], Travis J. Armiger[b], Li Lan[c], Kris Noel Dahl[a,b,*]

Supplemental Figures

Supplemental Figure 1.

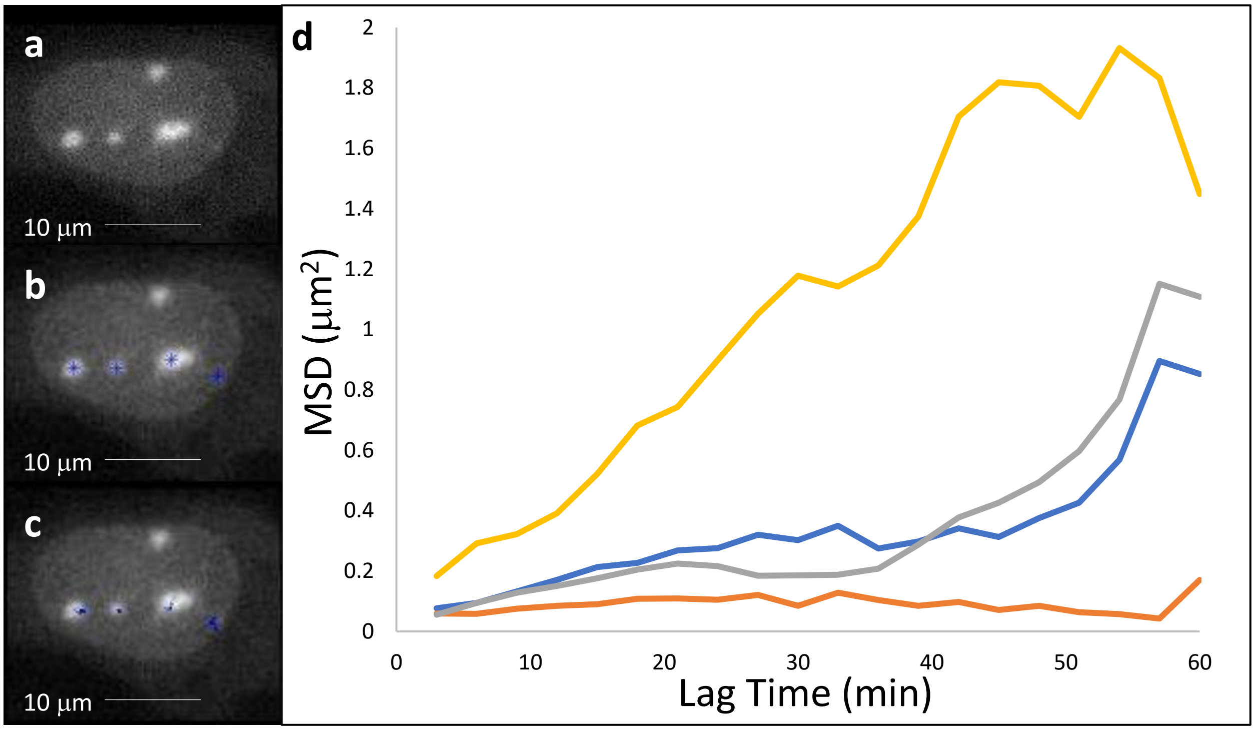

Supplemental Figure 1: ***Images and a graph showing various stages of data analysis.*** *(**a**) Representative image showing a cell after cropping and alignment procedure. (**b**) The same cell after particles that persist through all frames (blue asterisks) were identified by the algorithm. (**c**) The same cell after those particles were tracked by the algorithm. (**d**) Plot showing the individual tracks of each particle plotted as Mean Squared Displacement (MSD) in μm^2 versus Lag Time in minutes.*

Supplemental Figure 2.

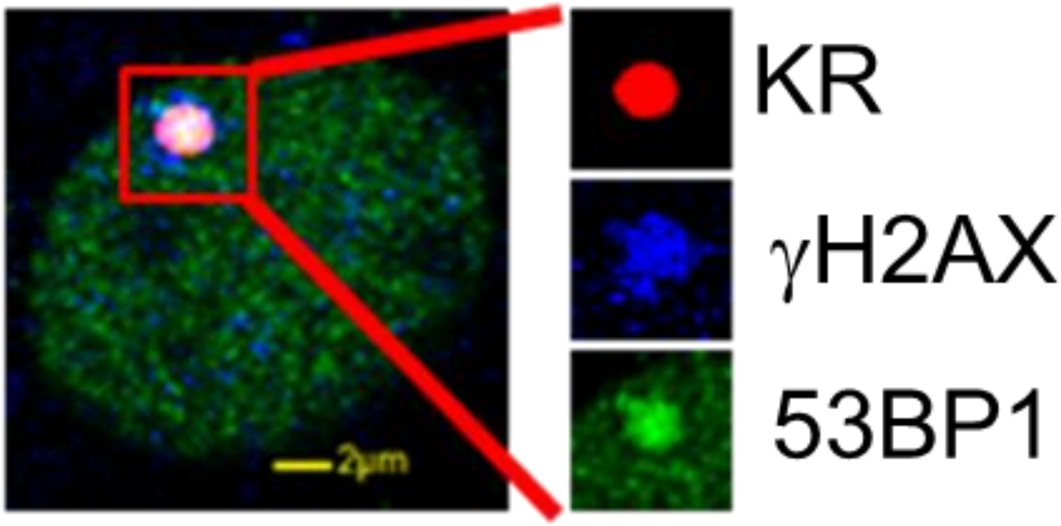

Supplemental Figure 2: ***Co-localization of TA-KR with DSB repair proteins.*** *U2OS cells were transfected with TA-KR and GFP-53BP1 and exposed to 10 min of white bulb light. Cells were then fixed with methanol-acetone (1:1) for 10 min at −20°C and then incubated with anti-γH2AX (1:400, Millipore 05636) in blocking buffer overnight at 4°C followed by Alexa Fluor 405 goat anti-mouse immunoglobulin G (Invitrogen). Cells were washed three times with PBST (PBS with Tween 20) buffer and incubated with Cell samples were then mounted in drops of PermaFluor (Immunon) and imaged at 60x 1.4 NA.*

Supplemental Figure 3.

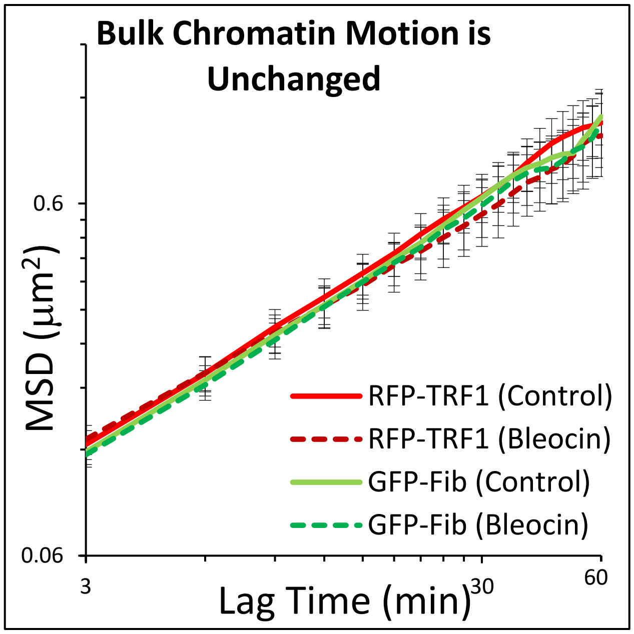

Supplemental Figure 3: ***Comparison of MSDs of two spatially and functionally distinct markers in U2OS cells under both control conditions and globally induced damage conditions.*** *All MSDs, regardless of probe or drug treatment are statistically indistinguishable. RFP-TRF1 (Control) (n=17) is shown as a solid red line. RFP-TRF1 (Bleocin) (n=18) is shown as a broken red line. GFP-Fibrillarin (Control) (n=13) is shown as a solid green line. GFP-Fibrillarin (Bleocin) (n=17) is shown as a broken green line. Error bars represent Standard Error of the Mean. RFP-TRF1 (Control) and GFP-Fibrillarin (Control) previously used in Figure 1c; RFP-TRF1 (Bleocin) and GFP-Fibrillarin (Control) previously used in Figure 5b.*

Supplemental Figure 4.

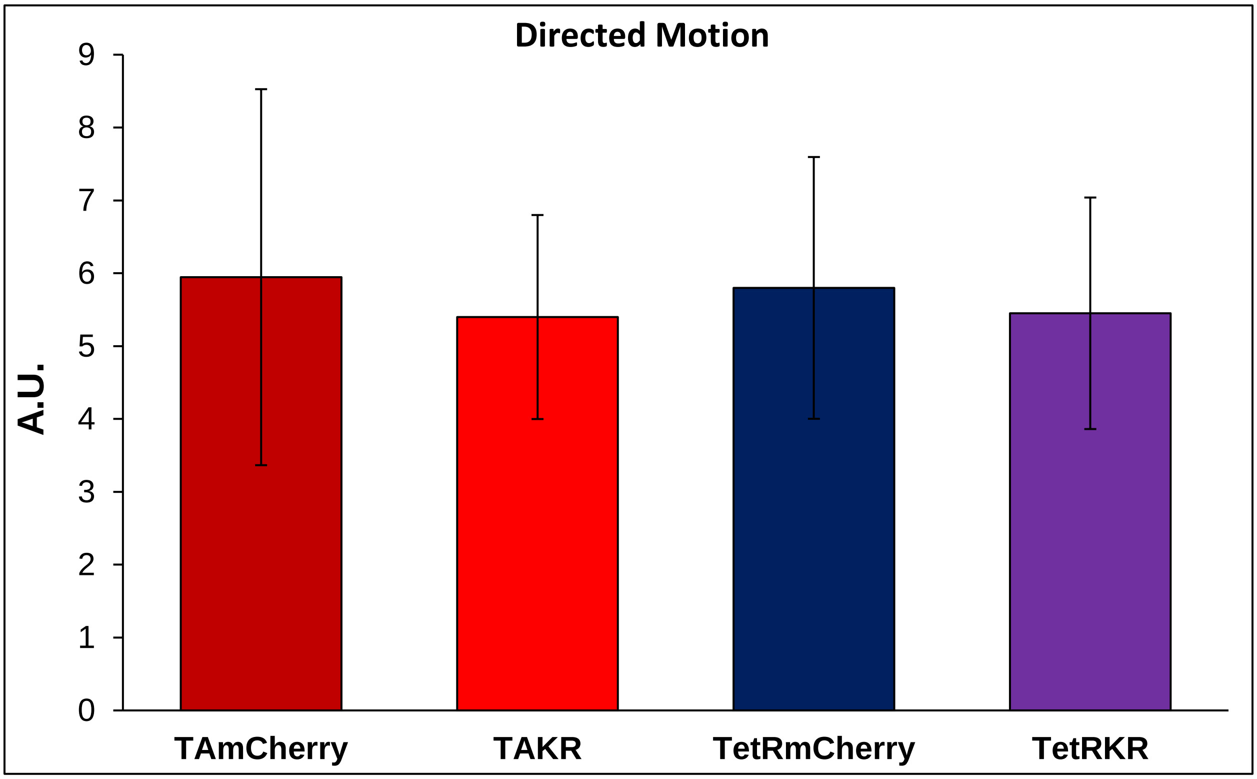

Supplemental Figure 4: ***Quantification of directed motion of the four TRE-bound tracers.*** *MSDs are inherently directionless, and the MSD of TAmCherry, TAKR, TetRKR statistically indistinguishable. Thus, an increase in the size of the region explored by the particles should indicate whether a particle is moving in a more directed way. Using particle tracking data, we determined the maximum range of both x and y axis motion to determine the diagonal of the resulting rectangle as this would be the maximum distance the particle could possibly have traveled during the experiment. There was no statistical difference between any of the samples ($p>0.5$ by Students t-test).*
